# A Shift to Human Body Temperature (37°C) Rapidly Reprograms Multiple Adaptive Responses in Escherichia coli That Would Facilitate Niche Survival and Colonization

**DOI:** 10.1128/JB.00363-21

**Published:** 2021-10-25

**Authors:** Anastasia Gant Kanegusuku, Isidora N. Stankovic, Pamela A. Cote-Hammarlof, Priscilla H. Yong, Christine A. White-Ziegler

**Affiliations:** a Department of Biological Sciences, Smith College, Northampton, Massachusetts, USA; b Program in Biochemistry, Smith College, Northampton, Massachusetts, USA; Brigham and Women’s Hospital/Harvard Medical School

**Keywords:** temperature, virulence, RpoS, stress response, biofilm, anaerobic respiration, transcriptional regulation, transcriptome, biofilms

## Abstract

One of the first environmental cues sensed by a microbe as it enters a human host is an upshift in temperature to 37°C. In this dynamic time point analysis, we demonstrate that this environmental transition rapidly signals a multitude of gene expression changes in Escherichia coli. Bacteria grown at 23°C under aerobic conditions were shifted to 37°C, and mRNA expression was measured at time points after the shift to 37°C (*t* = 0.5, 1, and 4 h). The first hour is characterized by a transient shift to anaerobic respiration strategies and stress responses, particularly acid resistance, indicating that temperature serves as a sentinel cue to predict and prepare for various niches within the host. The temperature effects on a subset of stress response genes were shown to be mediated by RpoS and directly correlated with RpoS, DsrA, and RprA levels, and increased acid resistance was observed that was dependent on 23°C growth and RpoS. By 4 h, gene expression shifted to aerobic respiration pathways and decreased stress responses, coupled with increases in genes associated with biosynthesis (amino acid and nucleotides), iron uptake, and host defense. *ompT*, a gene that confers resistance to antimicrobial peptides, was highly thermoregulated, with a pattern conserved in enteropathogenic and uropathogenic E. coli strains. An immediate decrease in curli gene expression concomitant with an increase in flagellar gene expression implicates temperature in this developmental decision. Together, our studies demonstrate that temperature signals a reprogramming of gene expression immediately upon an upshift that may predict, prepare, and benefit the survival of the bacterium within the host.

**IMPORTANCE** As one of the first cues sensed by the microbe upon entry into a human host, understanding how bacteria like E. coli modulate gene expression in response to temperature improves our understanding of how bacteria immediately initiate responses beneficial for survival and colonization. For pathogens, understanding the various pathways of thermal regulation could yield valuable targets for anti-infective chemotherapeutic drugs or disinfection measures. In addition, our data provide a dynamic examination of the RpoS stress response, providing genome-wide support for how temperature impacts RpoS through changes in RpoS stability and modulation by small regulatory RNAs.

## INTRODUCTION

In the course of its existence, Escherichia coli may transit through many different environments with concomitant changes in temperature. A mammalian host typically offers a dramatically warmer environment than ambient settings, whereas a host fever response presents a further increased temperature challenge. As a result, bacteria must rapidly sense and respond to these temperature changes to maximize survival through swift adaptations in gene expression and protein activity.

Previous genome-wide studies from our laboratory and others have characterized gene expression changes regulated by temperature, revealing the capacity of this cue to coordinately regulate hundreds of genes (1–8). Our investigations using E. coli K-12 MC4100 demonstrated that 423 genes, approximately 10% of the genome, are temperature regulated in a comparison between cells grown for 8 to 10 generations at 37°C and 23°C (6, 7). In this thermally long-term-adapted state at 37°C, genes associated with nutrient acquisition and utilization (carbohydrate, amino acid, and iron) were preferentially expressed at host temperature, indicating a role for human body temperature in modulating genes that are helpful for niche colonization (6). At 23°C, approximately 40% of the genes preferentially expressed are RpoS-controlled genes, broadly supporting and expanding the model that low temperature is a primary environmental cue that triggers the general stress response and governs biofilm formation (7).

Temperature control of gene expression is mediated by both global regulators and operon-specific mechanisms (reviewed in references 9 and 10). In our previous genome-wide studies, two prominent global regulators contributed to thermal adaptation: H-NS and RpoS. The histone-like nucleoid structuring protein (H-NS), conserved among Gram-negative bacteria, regulates the transcription of many environmentally responsive genes (reviewed in references 11 and 12). H-NS is a silencer that binds to AT-rich DNA and primarily functions to inhibit the expression of genes acquired by horizontal gene transfer, thus explaining its emphasis in regulating genes involved in virulence and niche adaptation (reviewed in reference 13). Temperature regulation and H-NS have been studied in the control of numerous operons (14–27) and in our own microarray studies, where H-NS alters the expression of approximately 75% of temperature-regulated genes in E. coli K-12 (28).

The coordinate control of gene expression by temperature also occurs through the general stress response sigma factor RpoS and the small regulatory RNA (sRNA) DsrA. Through a temperature-dependent mechanism, the transcription of *dsrA* is increased at low temperature, and the DsrA sRNA interacts with the *rpoS* mRNA to alter its secondary structure. These structural changes allow more efficient translation of the *rpoS* mRNA (reviewed in references 29 and 30), leading to an increase in RpoS production during low-temperature, exponential growth (31). Our observations that nearly half of the genes with increased expression at 23°C were previously shown to be RpoS dependent provide experimental evidence that supports this model during long-term adaptation to low-temperature growth (7).

In this report, we investigated the effect of a temperature shift from 23°C to 37°C, mimicking the temperature transition experienced by a microbe as it enters a human host. Unlike most previous studies, this strategy allowed the exploration of the kinetics of gene expression changes and the suite of genes required to respond to this environmental change within the first few hours after the temperature upshift. We demonstrate that a significant number of genes are rapidly altered in expression within minutes to hours after a temperature shift. Some are altered only transiently, while others overlap our previously identified genes that are required for long-term adaptation to either temperature. Many of these genes are related to respiration pathways, stress responses, and immune evasion, suggesting that temperature may serve as a sentinel cue to predict and prepare for niche adaptation in the host.

## RESULTS

### Microarray design to identify temperature-regulated genes in E. coli K-12.

The goal of this study was to determine which genes are rapidly modulated in response to a shift from 23°C to 37°C, mimicking the transition experienced by a bacterium as it moves from the external environment and enters a human host. The temperature-dependent experimental growth conditions were designed to ensure that cultures were initiated and harvested in nutrient-replete, early to mid-exponential phase (*A*_600_ = 0.3 to 0.6) for all time points, including the 23°C starting culture (*t* = 0 h) and the incubation time points after the shift to 37°C (*t* = 0.5, 1, and 4 h). This strategy ensured that changes in gene expression could be directly attributed to the temperature shift and not to changes in nutrient levels or growth phase. Microarray analyses were completed by comparing the expression levels at each 37°C time point to that of the initial starting 23°C culture at time zero. Temperature measurements on the aliquots demonstrated that full equilibration to 37°C takes 17 ± 0.8 min.

Using a false discovery rate of 1%, 994 genes showed statistically significant gene expression changes of ±1.2-fold at one or more time points (994/4,401; 23%). In this study, the analyses were focused on the 344 genes whose expression was altered by ±2.0-fold at one or more time points, representing 8% (344/4,401) of the genome (see Table S1 in the supplemental material). In previous studies, we measured expression in thermally adapted cultures at steady state (i.e., after 8 to 10 generations at either 37°C or 23°C) under the same growth conditions (6, 7). A comparison between studies demonstrates a common set of 132 genes whose expression is thermoregulated immediately upon a temperature upshift and for long-term growth at a given temperature. In contrast, 212 genes show temperature-mediated fluctuations only upon the initial transition but not for long-term-adapted growth (Table S1).

### A shift to 37°C elicits a transient response toward anaerobic respiration.

**FIG 1:**
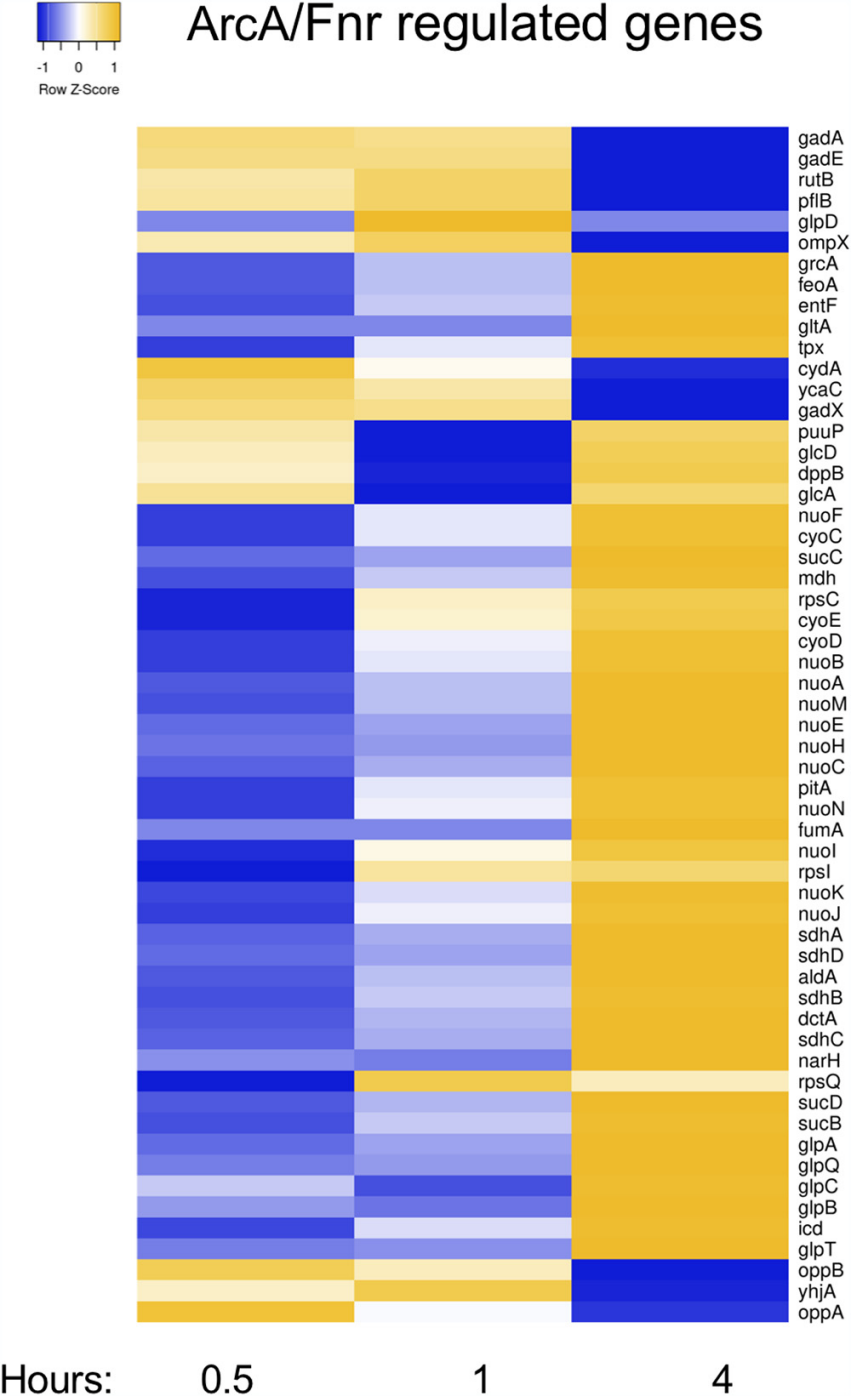
A shift in temperature to 37°C initially and transiently cues responses for anaerobic conditions primarily through the repression of genes associated with aerobic respiration at 0.5 and 1 h. The heat map illustrates the expression of ArcA/Fnr-regulated genes that showed a statistically significant change of 2-fold or higher at one or more time points during the time course experiment. Enrichment analyses (see Tables S1 to S4 in the supplemental material) support the overrepresentation of these genes at the first two time points in pathways associated with aerobic and anaerobic respiration, fermentation, and electron transfer and for the transcription factors ArcA and Fnr (Tables S2 to S4). The heat map was created using Heatmapper (100).

Upon the temperature upshift, genes primarily associated with aerobic respiration pathways show decreased expression at the 0.5- and 1-h time points, whereas no differential expression is observed at 4 h (Fig. 1). This initial shift to anaerobic respiration strategies occurs despite the cultures being aerated throughout the experiment. Enrichment analyses concur, showing a statistically significant overrepresentation of genes in pathways associated with aerobic and anaerobic respiration, fermentation, and electron transfer and for the transcription factors ArcA and Fnr that regulate the majority of these genes (Tables S1 to S4). Most differentially expressed genes in this subset (39/46) are negatively regulated by ArcA that represses the expression of aerobic respiration genes within the tricarboxylic acid (TCA) cycle, the glyoxylate shunt, electron transport chains, and fatty acid catabolism under microaerobic and anaerobic conditions (32, 33). A substantial subset of these ArcA-regulated genes are also negatively regulated by Fnr (34/46 Fnr-controlled genes) that decreases the expression of genes associated with aerobic respiration under anaerobic conditions, along with activating genes associated with anaerobic respiration and fermentation (32, 33). Thus, a shift in temperature to 37°C initially and transiently cues adaptive responses for anaerobic conditions primarily through the repression of genes associated with aerobic respiration. Four hours after the shift, however, this differential gene expression resolves, with the expression of these genes returning to levels similar to that of the initial 23°C aerobically grown culture. These results argue that a temperature upshift triggers a transient response favoring anaerobic respiration and agree with previous studies showing that temperature serves as a predictive signal for entry into the host environment (34) (see Discussion).

At the 4-h time point, some nontransient, longer-term, and likely adaptive responses were characterized by an overrepresentation of pathways predominantly associated with amino acid and nucleotide synthesis, which showed 2- to 5.3-fold increases compared to the starting 23°C culture, signaling turnover to an emphasis on growth and replication at 37°C. Additionally, siderophore synthesis and iron uptake pathways, negatively regulated by Fur, showed increased expression (Tables S1 to S4).

### An RpoS-dependent general stress response is transiently elicited upon a switch to 37°C.

**FIG 2:**
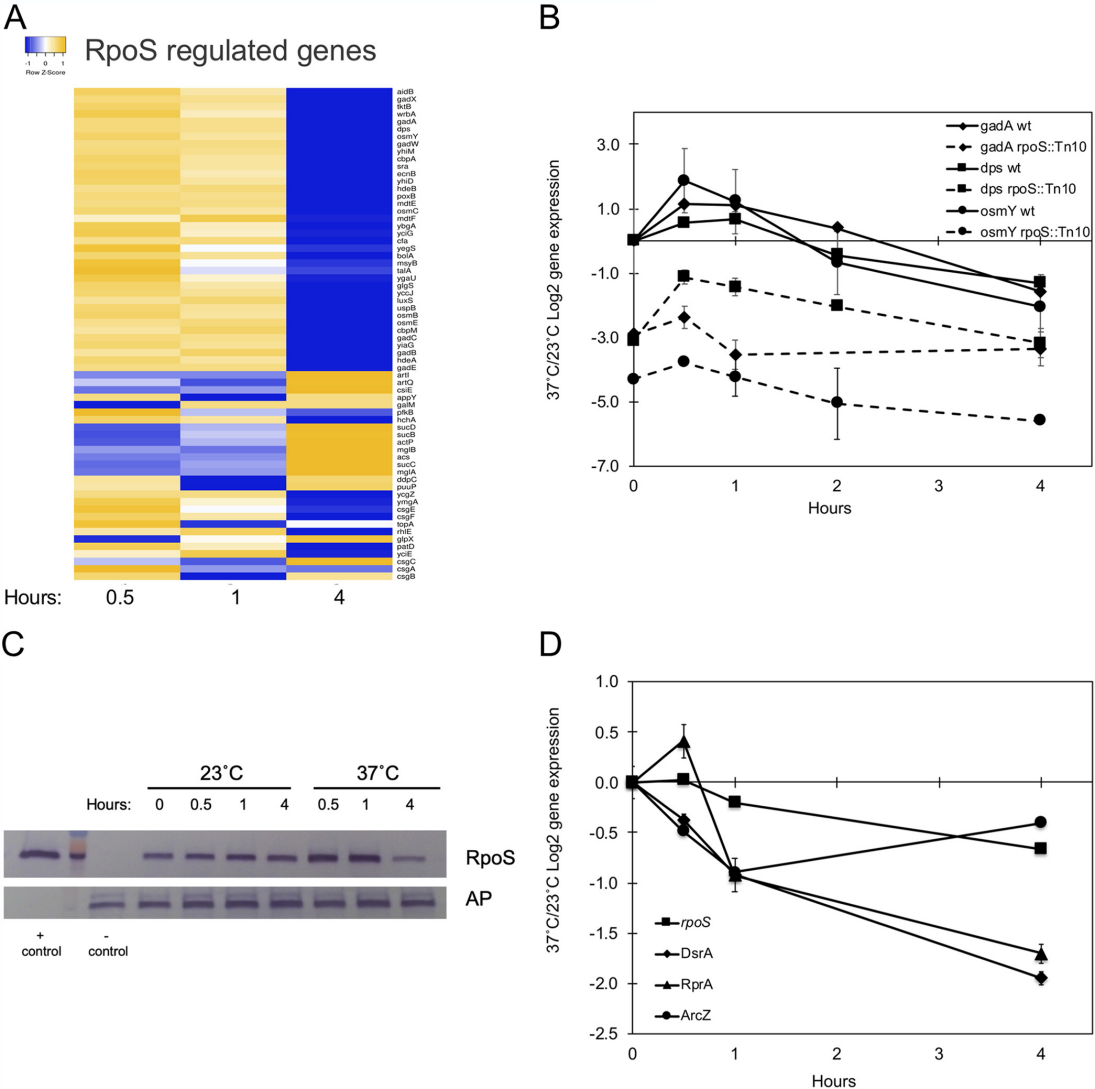
A shift to 37°C rapidly modulates general stress response gene expression, RpoS protein levels, and small regulatory RNAs that regulate RpoS expression. (A) Heat map illustrating the expression of RpoS-regulated genes that showed a statistically significant change of 2-fold or higher at one or more time points during the time course experiment. The heat map was created using Heatmapper (100). (B) qRT-PCR measurement of candidate stress response genes in the wild-type (wt) and *rpoS*::Tn*10* mutant strains at 37°C. Levels of expression are shown relative to the level of the starting 23°C wild-type culture. Data from a representative experiment are shown. (C) Western blotting of RpoS protein in cultures retained at 23°C and those shifted to 37°C. Alkaline phosphatase (AP) was used as a loading control. + control, purified RpoS protein; − control, total protein from the *rpoS*::Tn*10* insertion strain. (D) qRT-PCR measurement of sRNAs that regulate RpoS expression. Levels of expression are shown relative to the level of the starting 23°C wild-type culture. Data from a representative experiment are shown.

The expression of 66 genes known to be RpoS controlled under other environmental conditions (35) showed significant changes at 1 or more time points after a shift to 37°C. For these genes, we visualized two different patterns of expression over the time course of this experiment. The majority showed transiently increased expression at 37°C during one or both early time points (*t* = 0.5 and 1 h), which was subsequently reduced by the 4-h time point (Fig. 2A; Table S1). Significantly reduced transcription levels of three representative genes from this subset (*gadA*, *dps*, and *osmY*) in an *rpoS*::mTn*10* mutant background demonstrate that the thermoregulation of these genes is primarily RpoS dependent (Fig. 2B).

Further validating the RNA results, the expression pattern of these genes is matched by the RpoS protein levels during this transition. Immediately after a shift to 37°C, RpoS levels appear slightly elevated at the 0.5- and 1-h time points compared to 23°C, whereas RpoS is visibly decreased by 4 h (Fig. 2C), correlating the transient increase and the subsequent decrease in the expression of this subset of RpoS-dependent genes with RpoS levels. Our results concur with previous studies that demonstrated that heat shock at 42°C leads to the stabilization and increased half-life of RpoS through decreased proteolysis (36). Our time course extended longer than the experiments of Muffler et al. (4 h versus 40 min), allowing one to see that the increased stability of RpoS is transient, with levels dissipating by 4 h at 37°C.

To investigate whether the decreasing RpoS levels at 4 h correlate with the expression of small regulatory RNAs that regulate RpoS translation, the transcript levels of DsrA, RprA, and ArcZ were measured after the temperature upshift. DsrA and RprA levels were reduced 3.8- and 3.2-fold, respectively, by 4 h at 37°C (Fig. 2D). The temperature regulation of DsrA was previously established (31, 37), but to the best of our knowledge, this is the first instance where RprA has been shown to be thermoregulated. While ArcZ shows a transient dip, its expression and that of the *rpoS* mRNA are primarily unaffected by temperature (Fig. 2D). Thus, the continued expression of DsrA and RprA during the early time points may contribute to increased or sustained RpoS expression during an early shift to 37°C, and their disappearance correlates with the decreased RpoS levels by 4 h.

### Acid resistance genes controlled by RpoS are transiently and highly increased upon a temperature upshift.

Dominant among the RpoS-controlled controlled genes, in both number and increased expression, are genes associated with the acid stress response. These genes are initially increased by a shift to 37°C at the 0.5- and 1-h time points, followed by a subsequent drop in expression by 4 h. The majority of genes follow the early expression pattern of other RpoS-dependent genes, and in the case of *dps*, we have demonstrated that this thermoregulatory response is due to RpoS (Fig. 2B; Table S1). These include chaperones and DNA-protective proteins (*hdeAB*, *hch*, and *dps*), genes on the acid resistance island (*gadABC* and *mdtEF*), and other genes (*yhiM*, *yhiD*, and *yodD*) with reported roles in acid resistance (38–40). Previous studies have demonstrated that the simultaneous overexpression of DsrA, RprA, and ArcZ and their subsequent effect of increasing RpoS protein levels lead to increased acid tolerance during active cell growth (41). Our data parallel these results with high expression levels of these sRNAs and RpoS at 23°C and during the initial time points of the temperature upshift.

**FIG 3:**
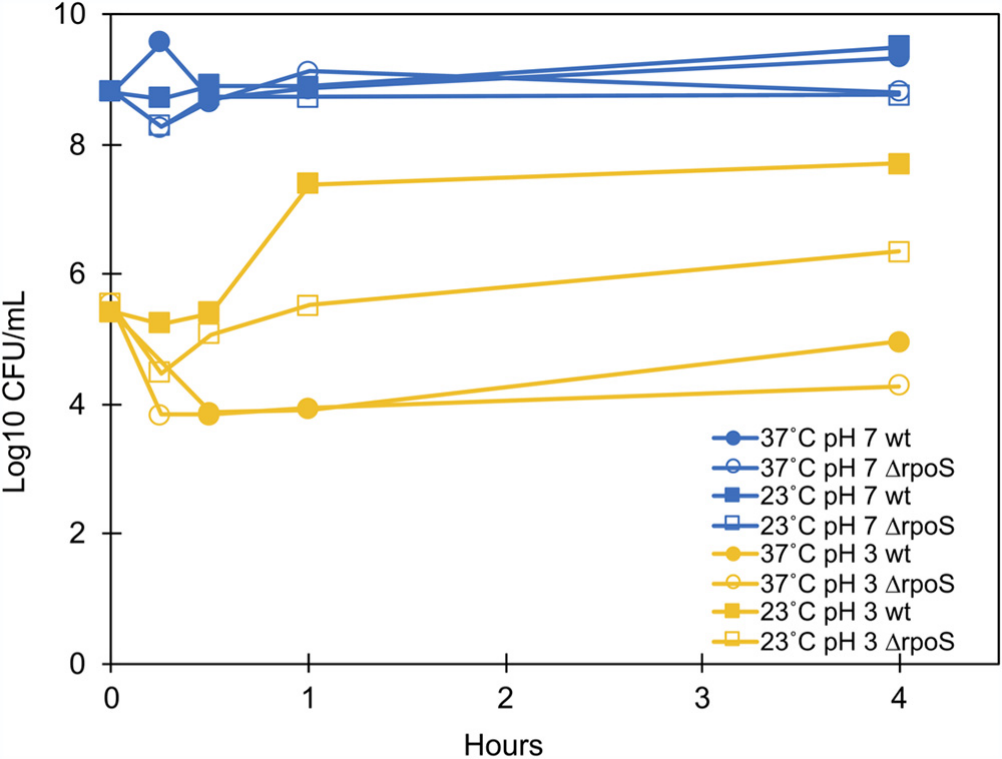
A shift to 37°C decreases the low-pH viability of the wild-type and *rpoS*::Tn*10* mutant strains in comparison to those grown at 23°C. Cultures were subjected to a decrease to pH 3 by the addition of HCl for 10 min at each time point, diluted, and plated at 37°C to determine the viable cell count. This assay was completed in triplicate.

To investigate if growth temperature altered the ability of cells to survive a low-pH challenge, cultures grown for various times at 37°C and 23°C were subjected to acid stress (pH 3), with the results that those grown at low temperature had increased resilience that was partially RpoS dependent. While the starting 23°C culture (*t* = 0 h) decreased 1,000-fold in viable cells per milliliter when the pH was lowered to 3, at subsequent time points the upshifted 37°C actively growing cultures demonstrated additional 10- to 1,000-fold reductions in viable cells counts in comparison to cultures retained at 23°C (Fig. 3). In comparison to the wild-type strain at 23°C, an *rpoS*::Tn*10* mutant demonstrated lower levels of viability, indicating that the temperature-dependent low-pH resilience at low temperature is partially due to RpoS. Interestingly, the wild type and the *rpoS*::Tn*10* mutant grown at 37°C were similarly susceptible to low pH at all time points. This would be expected at 4 h given that RpoS levels are decreasing along with the expression of acid resistance genes. However, the increased expression of acid resistance genes at the first two time points in our microarrays (*t* = 0.5 and 1 h) was predicted to confer some additional resistance to the wild-type strain compared to the mutant grown at 37°C.

### Curli and motility gene expression are conversely regulated by temperature.

A smaller subset of RpoS-dependent genes (14 genes) showed immediate, continued decreased expression throughout the time course. Particularly notable are the curli genes that demonstrated a rapid reduction (2- to 9.2-fold) within the first 30 min after a shift to 37°C. Curli expression, which facilitates biofilm formation (42), has previously been shown to be temperature regulated in E. coli K-12 (43), and our previous results have demonstrated that curli expression and biofilm formation are favored at lower temperature (7). While delayed in their response compared to the curli genes, other genes responsible for biofilm formation, *bolA*, *bdm*, the *ycgZ*-*ymgABC* operon, and the diguanylate cyclases *yedQ* and *ydaM* (44–47), are also influenced by temperature.

**FIG 4:**
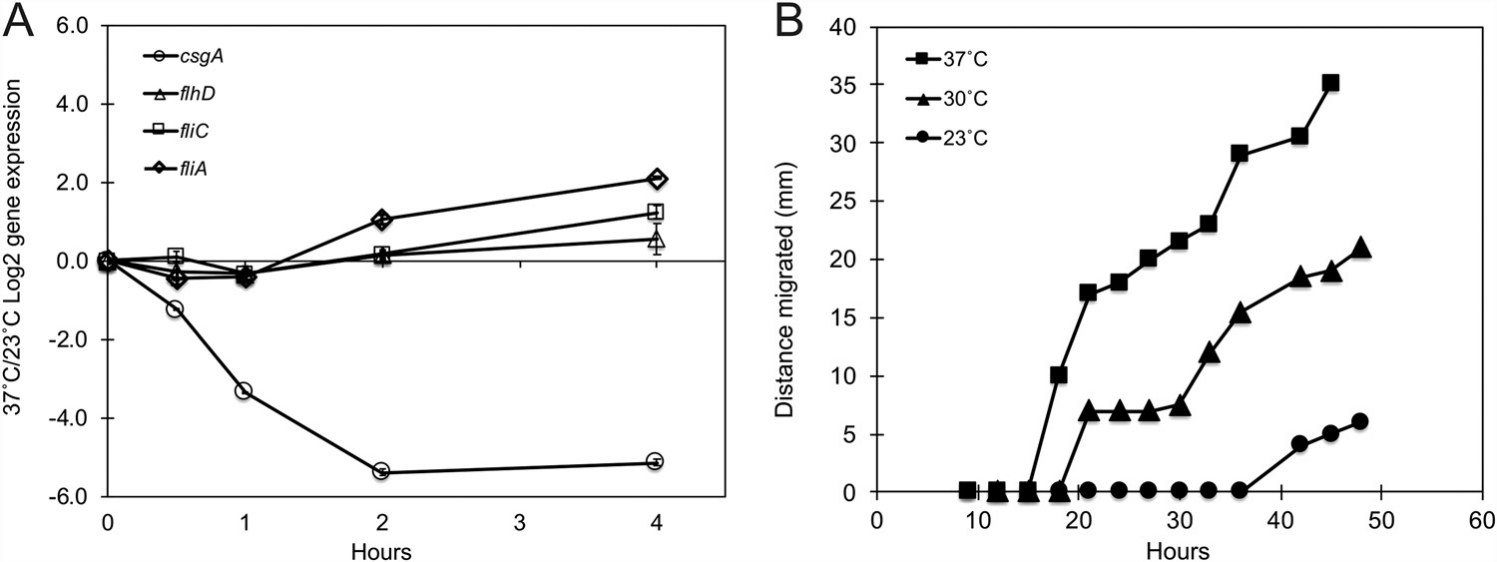
A shift to 37°C increases motility gene expression and motility in the wild-type strain. (A) qRT-PCR measurement of representative motility (*flhD*, *fliA*, and *fliC*) and curli (*csgA*) genes in wild-type strain MG1655 after a shift to 37°C. Levels of expression are shown relative to the level of the starting 23°C wild-type culture. Data from a representative experiment are shown. (B) Bacteria were inoculated onto M9 minimal glycerol solid medium with reduced (3.5%) agar, and the distance migrated was measured over time at three incubation temperatures (37°C, 30°C, and 23°C).

It has been well established that sessile biofilms and planktonic swimming cells represent two alternative modes of existence for E. coli, with the decision between them being mediated by environmental cues and transcriptional regulators. Given that a temperature upshift decreases curli gene expression and biofilm formation, the effect of temperature on motility gene expression was explored. Because the MC4100 strain that we utilized for the transcriptomic analyses is nonmotile due to a mutation in the master flagellar regulator FlhD (48), we assessed the effect of temperature on flagellar gene expression in the motile E. coli K-12 MG1655 strain using quantitative real-time reverse transcriptase PCR (qRT-PCR). While the expression of the master flagellar controller (*flhD*) was not impacted by temperature, genes for the flagellar sigma factor (*fliA*) and the main flagellar structural subunit (*fliC*) exhibited increased expression upon a shift to 37°C (Fig. 4A). In addition, assessment of motility over time at 37°C, 30°C, and 23°C demonstrated faster migration at 37°C than at lower temperatures (Fig. 4B). Together, these results support that changes in motility upon a temperature shift are due, at least in part, to changes in flagellar gene expression and that temperature is an environmental cue used in the developmental pathway for both biofilm formation and the planktonic state (see Discussion).

### Genes associated with host defense and iron utilization are more highly expressed at 37°C.

**FIG 5:**
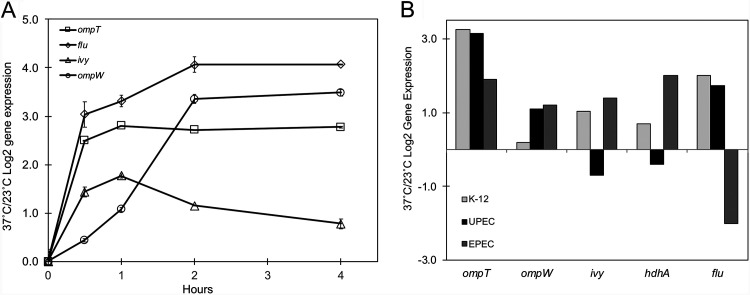
A shift to 37°C regulates virulence gene expression in K-12, uropathogenic, and enteropathogenic E. coli strains. (A) qRT-PCR measurement of virulence genes in the wild-type strain after a shift to 37°C. Levels of expression are shown relative to the level of the starting 23°C wild-type culture. Data from a representative experiment are shown. (B) Relative gene expression 4 h after a shift to 37°C in three strains of E. coli. K-12 microarray data are from this study; gene expression levels are from our unpublished transcriptome sequencing (RNA-Seq) experiments on the uropathogen E. coli CFT073 and the enteropathogen E. coli E2348/69.

Given that 37°C is a cue for entry into a human host and that many genes associated with virulence in pathogenic bacteria are thermoregulated (9, 10), we hypothesized that nonpathogenic E. coli might have similarly regulated genes. Multiple genes that contribute to host defense show increased expression upon a shift to 37°C in our microarrays, including *ompT*, *ivy*, *flu*, *hdhA*, and *ygiW* (Fig. 5B; Table S1). One of the most highly and rapidly thermoregulated genes in the entire transcriptome study encodes the outer membrane protease OmpT that has been shown to cleave antimicrobials, including cathelicidin (LL-37) (49); protamine (50); bacterial colicins E2, E3, and D (51, 52); and antimicrobial proteins isolated from human urine (53). Ag43 (*flu*) is known to promote autoaggregation, biofilm formation, and adhesion (reviewed in references 54 and 55). Its expression in intracellular uropathogenic E. coli (UPEC) biofilms and *in vivo* infection models and its prevalence in clinical urinary tract isolates all support the role of Ag43 in bacterial persistence (56–58). Studies in saliva, breast milk, and hen egg white indicate that Ivy, an inhibitor of vertebrate C-type lysozyme (59), promotes bacterial survival or growth (60). *ygiW*, the homologue of Salmonella enterica serovar Typhimurium VisP, is involved in the cellular response to hydrogen peroxide and cadmium stress in E. coli (40), and in Salmonella, a Δ*visP* mutant is attenuated for infection (61). HdhA is responsible for the catabolism of the bile acids cholic acid and chenodeoxycholic acid (62, 63) found in the host gastrointestinal tract. Using the more sensitive technique of qRT-PCR, the rapid and highly increased expression of several of these genes can be visualized in response to the host environmental cue of 37°C (Fig. 5A). Additionally, *ompW*, encoding a protein known to confer complement resistance through the binding of factor H (64), was confirmed to be highly regulated by temperature, confirming previous reports (6, 65).

As demonstrated in many bacterial pathogens (66–68), the ability to compete for and acquire iron is crucial for host infection. Similar to our previous study indicating that iron utilization genes are thermoregulated (6), in this study, we see that outer membrane transporters for iron uptake show increased expression at 37°C (*fepA*, *fecA*, *feoA*, *fhuA*, *cirA*, and *fiu*) along with *tonB*, encoding the central energy-transducing protein for multiple iron systems. Additional genes within the ferric (*fec*) and ferrichrome (*fhu*) uptake and enterobactin synthesis (*ent*) systems along with putative and cryptic iron transporter systems (*efeO*, *efeU*_2, *yddAB*, and *yncE*) also show high-temperature induction (69, 70, 101). Enrichment analyses support these observations, showing a statistically significant overrepresentation of 29 Fur-controlled genes with increased expression at 37°C (Tables S1 to S4).

Comparison of the regulation of these host defense genes in two pathogenic strains supports the importance of host temperature as a critical cue in virulence gene regulation. The uropathogenic strain CFT073 was grown under medium and growth conditions identical to those for our MC4100 strain, whereas the enteropathogenic strain E2348/69 was grown in Dulbecco’s modified Eagle’s medium (DMEM). Four hours after a shift to 37°C, *ompT* demonstrated highly increased expression in all three strains, and moderate increases in *ompW* expression were noted in UPEC and enteropathogenic E. coli (EPEC) strains (Fig. 5B). As in E. coli K-12, *flu* demonstrated increased expression at 37°C in UPEC, whereas its expression was decreased in EPEC. *ivy* and *hdhA* were more highly expressed in the EPEC strain at 37°C. It is interesting to note that while temperature impacts the gene expression patterns in these three strains, the thermoregulatory pattern itself is not always conserved.

### Gene expression of proteins associated with the outer membrane is altered with a change in temperature.

In addition to the outer membrane virulence proteins, porin gene expression changes with temperature. Increased *ompC* expression coupled with decreased *ompF* expression characterizes the initial upshift to 37°C and concurs with previous studies demonstrating that these porins are regulated by multiple environmental cues, including temperature (reviewed in references 71 and 72). By t = 4 h, however, thermoregulation is not observed for these porins. EnvY, a DNA-binding transcriptional regulator that participates in the thermoregulation of OmpC and OmpF expression as well as other cellular envelope proteins (73), is also highly thermoregulated, with a 9.3-fold increase at 37°C. While the expression of the larger porin OmpF is thought to allow greater access to nutrients, the expression of the smaller porin OmpC hampers the entry of deleterious compounds and is associated with resistance to antibiotics (74, 75). Also, porins serve as a site for colicin binding (76) and phage attachment (77, 78) and can be targets for immune responses by the host (79). Based on structural similarities, NmpC has been classified within the general bacterial porin family (80) and shows channel conductance (81). In our study, it is one of the most highly expressed genes (16.5-fold increase by 4 h) at 37°C. While thought not to be expressed in E. coli K-12 due to an IS*5* insertion (81), NmpC has been shown to contribute to increased heat resistance in E. coli strain AW1.7 (82) and increases sensitivity to several colicins and phages (83). Taken together, balancing the needs of the cell may illuminate temperature regulation, where changing porin composition may be a protective response to conditions in the host at 37°C, particularly within the intestine, where the cell encounters many challenges such as high osmolarity, bile, bacteriophage, and colicins along with the potential for antibiotics.

## DISCUSSION

In this study, we have demonstrated that a large number of genes, approximately 8% of the genome, are differentially expressed when E. coli K-12 is shifted to human body temperature. Measurable changes in gene expression are rapidly initiated within 30 min, while others show a more delayed response. The temperature-induced changes, whether transient or sustained, impact large subsets of genes that would facilitate microbial survival to host environmental conditions, suggesting that temperature may serve as a predictive cue to preemptively program adaptive responses.

**FIG 6:**
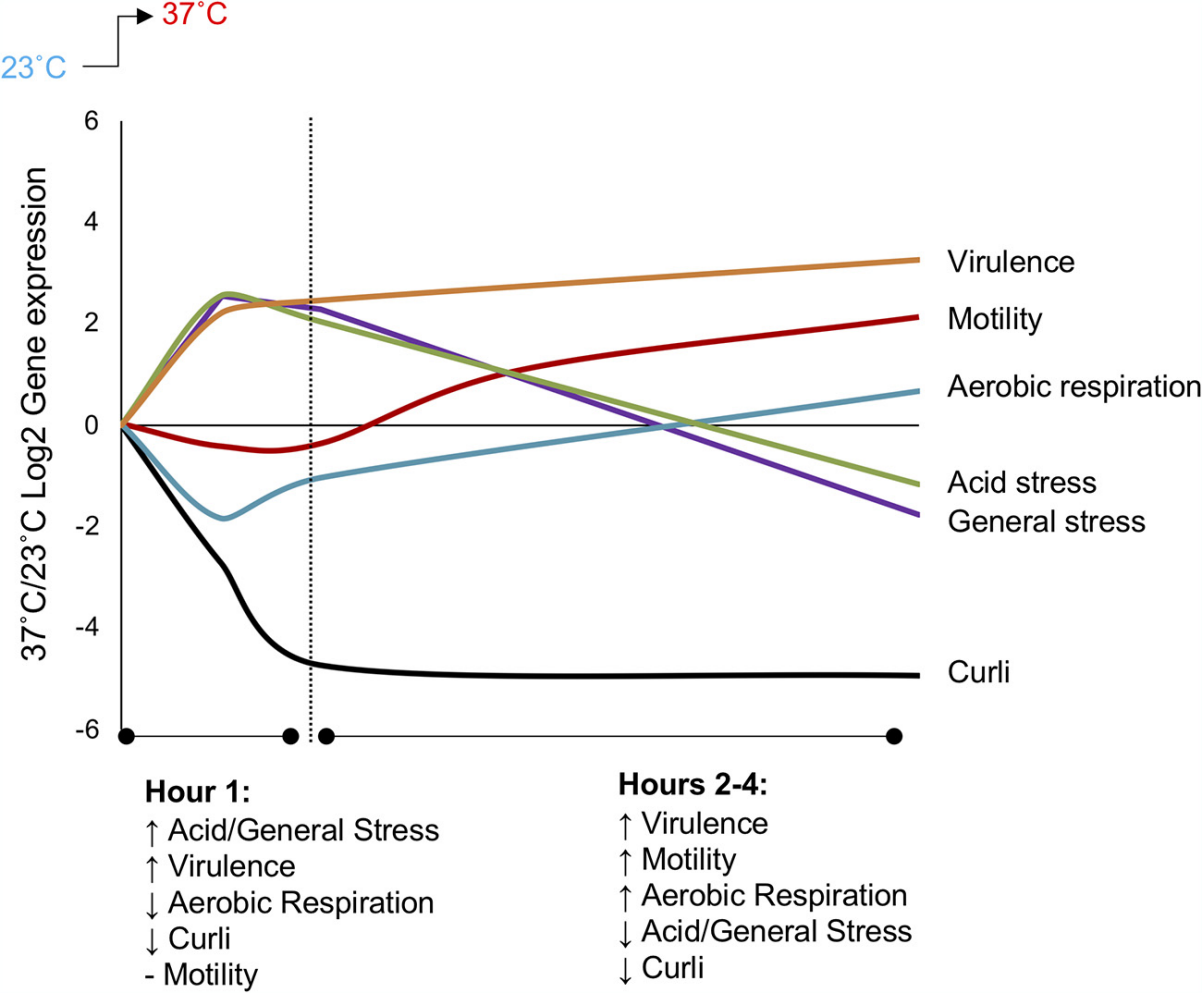
Model of the overall pattern of gene expression changes during the first 4 h after a temperature shift from ambient (23°C) to human host (37°C) temperature. The lines for each subset of genes represent the primary pattern of regulation observed for that group.

In our study, the dynamic patterns of gene expression changes cued within the first hour by a temperature upshift are ones that would facilitate adaptation to low oxygen, low pH, and other stressful conditions in the gastrointestinal tract of the host (Fig. 6). Genes associated with low-pH resistance are immediately increased, while genes associated with aerobic respiration are repressed, favoring anaerobic respiration strategies. In addition to low-pH resistance, other RpoS-mediated general response genes are increased that would confer resistance to other stressors (i.e., high osmolarity, oxidative stress, and high temperature) in various niches in the human body. Four hours after the temperature upshift, transcriptional responses move back toward aerobic metabolism and biosynthesis, whereas RpoS-dependent stress responses decrease, suggesting that reflexive responses to the actual culture conditions (oxygen replete, neutral pH, and higher growth rate) now dominate.

Our results correlating a temperature upshift with a shift to anaerobic respiration confirm and extend the dynamic predictive behavior characterized by Tagkopoulos et al. (34). In their study, they used transcriptional profiling to show that gene expression changes in response to a temperature upshift (37°C), even under highly aerobic conditions (18% oxygen), are highly correlated with those that occur in response to an oxygen downshift. Similarly, our cultures were continually aerated and demonstrated an equivalent response over a matching time frame (44 min versus 1 h). These authors hypothesized that the tight coupling of a temperature upshift to a preprogrammed anaerobic response would rapidly facilitate adaptation by using the immediate cue of host temperature to signal adaptive responses for the oncoming anaerobic environment of the gut. Our longer-term experiment indicates that this response is transient because by the 4-h time point, there is no longer a bias toward anaerobic respiration, suggesting that the microbe is preprogrammed for anaerobic respiration but over the longer term can sense and utilize cues to appropriately adapt to culture conditions. Future studies are needed to evaluate the mechanism of this tight coupling between temperature and anaerobic respiration.

Mechanistically, our study indicates that the dynamic changes of RpoS-mediated gene expression are primarily mediated through known temperature effects on RpoS stability and expression, potentially conferring broad stress resistance to the organism upon its entry into a host. Low-temperature growth leads to high levels of RpoS expression through small RNAs, as previously demonstrated for DsrA (29, 31), but our additional observation that RprA is temperature regulated indicates that it may contribute as well. The initial high levels of these sRNAs coupled with the initial stabilization of RpoS upon the temperature upshift from 23°C to 37°C lead to transiently high RpoS levels during this transition that mimics host entry. One can envision that this “stress-ready” position would be favorable for adapting to a broad set of inhospitable environments within the host. Indeed, our experiments showing increased resilience to low pH in cultures grown at 23°C support this hypothesis. We note that our *in vitro* studies demonstrate that the RpoS-mediated stress response attenuates by 4 h at 37°C, during which our experiments retained consistent environmental conditions (i.e., neutral pH, available nutrients, low-density growth, and aeration). This does not mimic the various niches in the human host, where we hypothesize that other environmental cues would perpetuate additional stress-specific regulatory mechanisms that would either retain a high level of RpoS or lead to the induction of RpoS-independent stress response strategies.

In addition to these stresses, our results demonstrate that temperature is an important cue for initiating important immune defense and host colonization factors. To our knowledge, this is the first report of thermal control for *ivy*, *flu*, and *ompT*, while previous studies from our laboratory and others have characterized the temperature control of iron acquisition genes recapitulated in this study that is Fur independent (6). Furthermore, our data coincide with various human and animal *in vivo* infections that have demonstrated the importance of these factors during infection. Thus, thermal regulation is advantageous as it directs energy saving toward the efficient control of the expression of these bacterial proteins primarily in a host setting. Transcriptome experiments under way in our laboratory in uropathogenic and enteropathogenic strains of E. coli concur, with both pathogenic strains showing a broad role for temperature in the control of multiple virulence genes (our unpublished data).

Given its role in thermoregulation, we predicted that we would see an increase in H-NS negatively regulated genes upon the upshift to 37°C. However, statistically significant enrichments occurred only at the latter two time points and only for a small subset of genes, most of which were RpoS dependent. This suggests that the combined regulatory mechanisms of derepression by H-NS and activation by operon-specific activators may take longer than the 4-h time course of this experiment.

Bacteria alternate between living in sessile, biofilm communities and living as planktonic, single cells (reviewed in reference 84), and our data indicate that temperature is an important cue that drives this decision. RpoS is responsible for driving the transcription of genes necessary for biofilm production, including curli, cellulose, and diguanylate cyclase genes (reviewed in reference 85). Given the temperature-dependent changes in RpoS expression described above, a gradual decrease in the expression of curli genes, along with others important for biofilm formation, was predicted. However, the quick and large reduction of *csg* transcript levels that outpaced the other RpoS-dependent genes indicates that an additional thermoregulatory mechanism is involved. Regarding motility, to our knowledge, this is the first report that flagellar gene expression (*fliA* and *fliC*) is temperature regulated in E. coli. In Listeria monocytogenes, a protein thermosensor has been identified that increases flagellar gene expression at 37°C (86), and it would be interesting to understand if a similar mechanism is at play in E. coli.

In addition to RpoS, we noted that the expression of several thermoregulated genes is also controlled by sRNAs, raising the question of whether temperature control of sRNA expression may play a broader role in temperature-controlled gene expression. For example, the small regulatory RNAs *omrA* and *omrB* have been shown to negatively control the expression of *ompT* and *flu*, two genes that we have shown to be thermoregulated (87, 88). Preliminary studies in our laboratory have indeed shown that several sRNAs are thermoregulated, and studies are under way to determine their causal role in temperature regulation.

Together, the results of this study define how temperature cues gene expression changes that would contribute to bacterial resilience and adaptation in the host. External to a host, low-temperature external ambient settings lead to high expression levels of RpoS-dependent genes such that the microbe enters the body ready for a broad set of environmental stressors. This response is transiently enhanced upon entry into the host at 37°C, while at the same time, additional strategies are being induced to increase immune evasion, motility, biosynthesis, and appropriate respiration strategies. Beyond our understanding of bacterial physiology, these insights into the various pathways of thermal regulation could yield valuable anti-infective targets for chemotherapeutic drugs that would decrease the ability of bacteria to compete and survive within the host or for disinfection strategies in external biomedically relevant settings.

## MATERIALS AND METHODS

### Strains and media.

The strains used in this study include DL1504, an MC4100 Δ*lacZYA* strain (89); DL3106, an MC4100 Δ*lacZYA rpoS*::Tn*10* strain (7), and AAEC198A, a motile MG1655 Δ*lacZYA* strain (90). M9 minimal (M9) and Luria-Bertani (LB Lennox) media were prepared as described previously (91, 92).

### Bacterial growth conditions.

Bacterial cultures were inoculated and grown in M9 glycerol (M9 minimal liquid medium containing 2.45 μM ferric citrate, 30 μM thiamine, 100 μM calcium chloride, 1 mM magnesium sulfate, and 0.2% glycerol as a carbon source [pH 7]) with aeration at 225 rpm as described previously (6, 19). For the temperature shift, individual colonies were used to initiate a 20-ml culture. This initial culture was grown to mid-exponential phase (*A*_600_ = 0.2 to 0.6) and used to inoculate a larger, 200-ml culture that was grown to early to mid-exponential phase (*A*_600_ = 0.2 to 0.6) at 23°C. This culture was diluted 1:2 or 1:3 in fresh M9 minimal glycerol medium and grown for an additional 120 min at 23°C to early exponential phase (*A*_600_ = 0.15 to 0.2). At the initiation of the temperature shift, aliquots of the culture were retained at 23°C (control) or shifted to 37°C for various times (*t* = 0.5, 1, and 4 h). Because growth rates in M9 minimal glycerol medium differ by 3.1-fold between these two temperatures (37°C, 1.8 ± 0.1 h; 23°C, 5.6 ± 0.2 h), the 1- and 4-h cultures at 37°C were further diluted with fresh medium at the time of the shift to 37°C to ensure that cells harvested at all time points were in early to mid-exponential phase (*A*_600_ = 0.3 to 0.6).

For growth curve analyses, an initial culture was inoculated as described above and grown at 23°C to early exponential phase. The culture was subsequently shifted to 37°C, and spectrophotometer readings were taken at time points after the shift. The generation time for each temperature was determined as described previously (91).

### RNA isolation.

For microarray analyses and qRT-PCR experiments, RNA was isolated by phenol-chloroform extraction, followed by RNeasy column purification (Qiagen) and two DNase digestions, as described previously (6). Isolated RNA for microarray analyses was further concentrated using RNeasy MinElute columns (Qiagen). RNA concentrations and purity were determined by Nanodrop spectrophotometer readings. Isolated RNAs were stored at −20°C until use.

### cDNA synthesis, labeling, and hybridization.

Synthesis, labeling of cDNA with Cy3/Cy5, and hybridization were performed using the 3DNA array 350 RP expression array detection kit (Genisphere) as described previously (6). cDNA for each condition was prepared from 3.0 μg total RNA. cDNA was cohybridized to slides containing full-length PCR products from all 4,290 annotated open reading frames (ORFs) in E. coli MG1655. Slides were produced by the University of Alberta and obtained at a reduced cost through the Genome Consortium for Active Teaching (https://bio.davidson.edu/gcat/). Each slide contained three printings of the PCR products, yielding three technical replicates on a given slide. Three independent biological replicates were completed for the time course experiment. cDNA from each 37°C time point was cohybridized with cDNA from its corresponding 23°C starting culture at 0 h.

### Microarray data analysis.

Hybridized slides were scanned using GenePix Pro 4.1 software (Axon Instruments, Inc.). The photomultiplier tube voltage was altered for the initial scanning to ensure proper channel balance and decrease background. Any gene feature that had a signal-to-background noise ratio of <2.0 was rejected and not further analyzed. The ratio of median value data set for each array was then uploaded to the Babelomics 4 gene expression and functional profiling analysis suite (93) for normalization between experiments and imputation of missing values. Significance analysis was completed as a one-class response using Significance Analysis of Microarrays (SAM) (94) with a Δ value of 1.68 and a median false discovery rate of ≤1% for all time points. The full genome gene list for MG1655 was retrieved from EcoGene 3.0 (95). Enrichment analyses for pathways, transcriptional/translational regulators, and Gene Ontology (GO) biological processes were completed using the default parameters in EcoCyc (96). Genes were matched to their regulatory transcription and/or sigma factors using the RegulonDB database (97).

### qRT-PCR.

Reactions were completed using the SYBR green one-step qRT-PCR kit (Invitrogen) as described previously (6). All reactions were performed in triplicate, with no reverse transcriptase controls run for each RNA sample to detect DNA contamination. All reactions were normalized by using the same amount of total RNA (50 ng) for each reaction. Relative levels of gene expression and error analyses were calculated as previously described (6, 98) and shown relative to the 23°C starting culture at 0 h.

### Immunoblotting.

Western blotting was performed as described previously (99). Protein concentrations were determined by a bicinchoninic acid (BCA) assay, and equal amounts (5 μg) of total cellular protein were analyzed by SDS-PAGE and transferred to nitrocellulose. Detection of RpoS was completed by utilizing a monoclonal antibody (clone 1RS1, catalogue number W0009; BioLegend) at a dilution of 1:1,000 and horseradish peroxidase (HRP)-conjugated secondary goat anti-mouse IgG (Promega) at a dilution of 1:2,500. Detection of alkaline phosphatase was completed by utilizing a rabbit polyclonal antibody (catalogue number PA1-26208; Thermo Fisher Scientific) at a dilution of 1:15,000 and HRP-conjugated secondary goat anti-rabbit IgG (Thermo Fisher Scientific) at a dilution of 1:2,500. Purified RpoS protein (catalogue number CP009; Neoclone) was used as a positive control. Signal detection was completed using a 3,3′,5,5′-tetramethylbenzidine peroxidase substrate detection system (KPL).

### pH stress viability test.

Bacterial cultures were grown as described above for temperature shift experiments with 20-ml aliquots of the culture grown at 23°C (control) or shifted to 37°C for various times (*t* = 0.5, 1, and 4 h). At each time point, the cultures were subsequently split: 10 ml served as a control retained at pH 7, while the remaining 10 ml was reduced to pH 3 using HCl. The flasks were incubated for 10 min under their original temperature and aeration conditions. After incubation, 100 μl was removed from each condition and diluted 1:2 in a 96-well microtiter plate. Serial dilutions (1:10) were performed, and 10 μl of each dilution was plated onto M9 minimal glycerol agar plates and incubated for 24 to 36 h at 37°C. Colonies were counted to determine the viable cell count. Assays were performed in triplicate.

### Motility assay.

Assays were completed using M9 minimal glycerol solid medium with a reduced (3.5%) concentration of agar. A single colony was used to inoculate a 5-ml culture incubated at 37°C for 12 h with aeration. Cultures were subsequently diluted to early exponential phase (*A*_600_ = 0.1 to 0.2). The agar plates were needle inoculated using a liquid culture, and the temperature was shifted to 23°C, 30°C, and 37°C. Measurements of the swarm ring diameter were recorded over time.

### Data availability.

The GEO accession number for the microarray data reported in this paper is GSE165794.
